# Serum Albumin Trends in Relation With Prognosis of Individuals Receiving Hemodialysis Therapy

**DOI:** 10.7759/cureus.19958

**Published:** 2021-11-28

**Authors:** Gulsah Boz, Koray Uludag

**Affiliations:** 1 Nephrology, Kayseri Training and Research Hospital, Kayseri, TUR

**Keywords:** hemodialysis, trajectory, joint model, serum albumin, all-cause death

## Abstract

Introduction

Hypoalbuminemia is recognized as an indication of protein-energy depletion in several disease states. According to many studies, hemodialysis (HD) patients who have decreased baseline serum albumin levels exhibit a poor prognosis. However, serum albumin does not stay at a constant level with the progress of the disease, considering that only a baseline value may not precisely reflect prognostic value. The study objective was to ascertain whether there is a link between serum albumin trajectories and all-cause mortality in incident HD patients.

Methods

Retrospective cohort analysis was conducted in the HD unit at the University of Health Sciences, Kayseri Training and Research Hospital, Nephrology Clinic between June 19, 2010, and December 29, 2017. A total of 408 individuals aged 18 years or older, who had at least one measurement of serum albumin at baseline, were enrolled. The outcome was all-cause death. Time-dependent Cox regression and joint model were used to investigate the associations between serum albumin trend in time and the risk of all-cause mortality.

Results

Mean (SD) age was 62.17 (12.33) years, and 50.7% were male. At baseline, the mean (SD) albumin level was 3.59 (0.27). A faster decrease (per 1-SD increase in negative slope) in serum albumin levels was associated with increased risk of all-cause mortality (HR, 1.63; 95% CI, 1.08-2.84; p=0.023) in a fully adjusted joint model with slope parameterization. Also, an annual 1-SD increase in albumin level declined the hazard of all-cause mortality by 22% (HR, 0.78; 95% CI, 0.66-0.92; p=0.008) in a fully adjusted joint model with value parameterization. Similar results were obtained from time-dependent Cox models.

Conclusion

These findings suggest that longitudinal albumin evaluation, including the rate of change as a slope parameter, may be valuable for risk stratification of patients receiving HD.

## Introduction

The prevalence of protein-energy wasting (PEW) combined with inflammation as relevant prognostic factors are high in hemodialysis (HD) patients, ranging from 18%-75% across observational studies [1,2]. Earlier studies also reported that serum albumin was positively correlated with PEW indicators such as mid-arm circumference, lean body mass, and subjective global assessment score in HD patients [3,4], as in patients with chronic kidney diseases [5,6]. On the other hand, established risk factors for cardiovascular disease alone could not address the elevated death rates in these patient populations [7,8]. In this context, serum albumin concentration has also been shown to be an independent marker in predicting mortality in patients with end-stage renal disease (ESRD) [9-12].

Evidence is scarce on the relationship between the temporal evolution of serum albumin levels and survival in HD patients. Some studies reported that time-varying levels of albumin using averages, trimonthly alterations, or measurements in limited periods had a prognostic value as in baseline levels [13-15]. However, the time-varying trend could not be adequately addressed by evaluating only baseline exposure measurements or re-measuring the predictor after a first follow-up time frame only, that is, not at periodic intervals during the course of a longer-term follow-up, or using the difference between two measures for disease outcome. Furthermore, analysis using the time-dependent Cox model would yield biased predictions of biomarker impact, distorting their true explanatory power [16-20]. No study so far has assessed the association between the speed of change in albumin level and mortality risk in HD patients. We, therefore, aimed to assess whether serum albumin changes over time, as level or slope (change rate) parameter, are predictors of survival independent of other covariates in HD patients by modeling longitudinal and survival data simultaneously.

## Materials and methods

Setting and patients

We conducted a single-center retrospective cohort analysis of participants receiving maintenance hemodialysis at least three times per week for three months at an outpatient dialysis facility in The University of Health Sciences, Kayseri Training and Research Hospital, Nephrology Clinic. The study began on the day of the initial clinic visit, represented as the first assessment date of laboratory data and dialysis prescription. Patients were observed with regularity at least once a month, depending on the patient's status. They were attended from June 19, 2010, to December 29, 2017, and followed for a total of eight years, or until an endpoint event occurred or data was censored due to loss to follow-up, migration to another facility, kidney transplantation, or the study's termination on January 1, 2019. The hospital's database was employed for collecting clinical and biochemical data from electronic records or patient folders. Individuals had to be 18 years old or older and to have a minimum of one serum albumin testing at commencement. We excluded individuals who had stopped hemodialysis within three months of starting treatment (n=26), had malignancy (n=19), or had cirrhosis of the liver (n=7). After excluding those subjects, a total of 408 patients remained for final analysis. The Ethics Committee of­­­­­ the hospital approved the study, conducting in agreement with the ethical standards specified in the Declaration of Helsinki.

Outcome, primary predictor, and covariates

The endpoint was all-cause mortality, defined as the time from the start of the study to dying. The serum albumin trajectory over time was the main predictor of interest. We measured the average albumin level at the commencement of the study (the first calendar quarter) and every quarter after that. Each patient's first (baseline) quarter was the calendar quarter in which the subject had been on dialysis for over three months. We determined the following demographic and clinical features at the baseline period: age, gender, dialysis vintage, vascular access form, and coexisting disorders. The body mass index (BMI) was figured out by dividing the post-hemodialysis bodyweight (kg) by the square of stature. Hemoglobin, ferritin, serum creatinine, single-pool Kt/V, albumin, C-reactive protein (CRP), phosphorus, and parathyroid hormone (PTH) were all tested in the central lab and registered at various time intervals throughout the research duration, once monthly (albumin, phosphorus, hemoglobin, and creatinine) and every three months (CRP, ferritin, and PTH). Blood was drawn before the dialysis session, except for the post-dialysis serum urea nitrogen used to detect urea kinetics.

Baseline age was used for analysis, and dialysis vintage was the duration between hemodialysis commencement and the date of censoring or death in each subject. Single-pool Kt/V for urea was determined as a dialysis dosage indication using the urea kinetic model. Hypertension was defined as a systolic blood pressure of 140 mmHg or higher/diastolic pressure of 90 mmHg or higher, or a pair of hypertension diagnoses and antihypertensive medication usage. Diabetes mellitus (DM) was diagnosed if antidiabetic medications were used, overnight fasting blood glucose was higher than 126 mg/dL, or HbA1C was higher than six percent. Cardiovascular disease was defined as a history of myocardial infarction, bypass surgery, coronary artery stenosis, heart failure, peripheral arterial disease, or cerebrovascular disease.

Statistical methods

Box plots and histograms were used to assess the spread of continuous variables. Continuous data with normal distribution were presented as a mean and standard deviation, whereas skewed distributions were displayed as a median and 25th-75th percentile. As appropriate, the chi-square test, a one-way ANOVA test, or the Kruskal-Wallis test were used to evaluate baseline disparities. Because CRP, PTH, ferritin, and dialysis vintage were positively skewed, they were scaled using their interquartile ranges (IQR) in all regression analyses so that a one-unit change corresponded to one IQR increase in its value, and predictions were generated using these models. A natural scale was used to model the remaining variables. Subjects were divided into tertiles for analysis using the gradient of blood albumin levels.

First, the hazard function of all-cause mortality was estimated with time-dependent Cox models, and the hazard ratios (HR), 95% confidence intervals, and p-values were obtained. Next, a joint longitudinal-survival model was used to evaluate the association between albumin trajectory and mortality risk. The method examines the impact of a biomarker's rate of change on the likelihood of developing an outcome. The first derivative of the link between albumin trajectories and the hazard ratio of the clinical outcome showed the relative change in the hazard of outcome for a 1-SD rise in albumin change rate (decrease rate) or 1-SD increase in albumin per year. These models have previously been thoroughly studied in the literature [21,22]. Statistical adjustments with baseline variables were as follows: (i) crude model; (ii) age and sex; (iii) BMI, hemoglobin, ferritin, and dialysis vintage (iv) creatinine, single-pool Kt/V, phosphorus, PTH, and CRP; (v) vascular access type, presence of DM, hypertension, cardiovascular disease (CVD), and other comorbidities. We also used the joint model's longitudinal and survival components to predict conditional survival expectations for two patients, intending to show graphical illustrations.

Multiple imputations using chained equations with 10 repetitions were also applied to overcome missingness in baseline data, avoiding patients from being ruled out owing to the missing data. Age, gender, single-pool Kt/V, BMI, hemoglobin, and comorbidities were the covariates without missing data in the model. Ferritin (n=2), phosphorus (n=5), PTH (n=23), and CRP (n=10) were the parameters that necessitated imputing. All analyses were carried out with the R statistical program and the "JMbayes2" and "MICE" packages (R Foundation, Vienna, Austria). All tests were two-tailed, with P values less than 0.05 deemed statistically meaningful.

## Results

Baseline and longitudinal characteristics

Four hundred and eight patients were included in the study. The overall population’s mean (SD) age was 62.17 (12.33) years, and 50.7% were male, with a mean (SD) baseline BMI of 25.32 (5.25) kg/m2. 36.3 percent of the subjects had DM diagnosis, 70.1% had HT, and 33.6% had CVD. At baseline, the mean (SD) albumin level was 3.59 (0.27). Table 1 presented the baseline characteristics and laboratory data of all 408 incident dialysis patients when beginning HD, categorized by tertiles of serum albumin gradients.

**Table 1:** Baseline characteristics of the study cohort according to overall and tertiles of slopes of serum albumin Normally distributed continuous variables are presented as mean (standard deviation), and non-normally distributed variables as median (25th–75th percentile). Categorical variables are presented as numbers and percentages. BMI - body mass index; AVF - arteriovenous fistula; PTH - parathyroid hormone; CRP - C-reactive protein; Kt/V - measurement of dialysis adequacy (K=dialyzer clearance [L/hour], t=time [hour], V=distribution volume of urea [L]). *The body-mass index is the weight in kilograms divided by the square of the height in meters.

	Overall	Tertile 1	Tertile 2	Tertile 3	p-value
		< -0.002	(-0.002, 0.002]	> 0.002	
n	408	136	136	136	
Age, years	62.17 (12.33)	65.04 (12.61)	61.12 (11.86)	60.37 (12.07)	0.003
Male, n (%)	207 (50.7)	71 (52.2)	70 (51.5)	66 (48.5)	0.814
Baseline BMI*, kg/m^2^	25.32 (5.25)	24.41 (5.38)	25.49 (5.27)	26.06 (4.99)	0.031
Dialysis vintage, years	4.10 [2.10, 7.43]	4.45 [2.25, 7.20]	4.10 [2.18, 8.20]	3.95 [2.00, 7.45]	0.720
Vascular access (AVF), n (%)	189 (46.3)	53 (39.0)	67 (49.3)	69 (50.7)	0.106
Comorbidities					
Diabetes mellitus, n (%)	148 (36.3)	41 (30.1)	50 (36.8)	57 (41.9)	0.129
Hypertension, n (%)	286 (70.1)	101 (74.3)	99 (72.8)	86 (63.2)	0.098
Cardiovascular disease, n (%)	137 (33.6)	49 (36.0)	47 (34.6)	41 (30.1)	0.565
Others, n (%)	42 (10.3)	13 (9.6)	15 (11.0)	14 (10.3)	0.923
Hemoglobin, g/dl	11.07 (2.07)	11.23 (2.23)	10.91 (2.04)	11.07 (1.94)	0.436
Ferritin, µg/L	287.30 [158.07, 485.50]	298.15 [161.80, 515.70]	276.15 [149.68, 462.92]	287.55 [169.25, 479.70]	0.499
Phosphorus, mg/dL	5.04 (1.60)	4.99 (1.67)	5.04 (1.68)	5.10 (1.44)	0.842
PTH, µg/L	216.50 [113.80, 408.45]	220.25 [119.07, 415.22]	191.00 [96.15, 409.20]	221.60 [107.08, 400.65]	0.704
Serum albumin, g/dL	3.59 (0.27)	3.40 (0.23)	3.60 (0.21)	3.77 (0.24)	<0.001
CRP, mg/L	5.40 [2.70, 9.62]	5.85 [3.18, 10.12]	4.95 [2.68, 9.33]	4.65 [2.48, 9.50]	0.218
Serum creatinine, mg/dL	7.60 (2.08)	7.55 (1.92)	7.73 (1.96)	7.52 (2.34)	0.669
Single-pool Kt/V	1.25 (0.18)	1.23 (0.18)	1.27 (0.20)	1.23 (0.18)	0.080

Four thousand and four hundred seventy-seven tests with a median count (25th, 75th percentile) of nine (5, 15.25) per patient, over a median (25th, 75th percentile) follow-up of 2.16 (1.13, 3.73) years, were used to assess albumin changes between the baseline and the end of the follow-up time. The albumin slope varied from -0.016 to 0.023 each year using the longitudinal part of the joint model. During the course of the study, 188 (46%) subjects died from cardiac (n = 95) issues, malignancy (n = 5), infection (n = 46), and unidentified reasons (n = 42). When transitioning from tertile one (rapid decliners) to tertile three of the serum albumin slope, there was a tendency of decreasing age and rising baseline BMI (Table 1). There were no differences in other baseline variables among tertiles.

Temporal patterns of albumin and mortality

**Figure 1:**
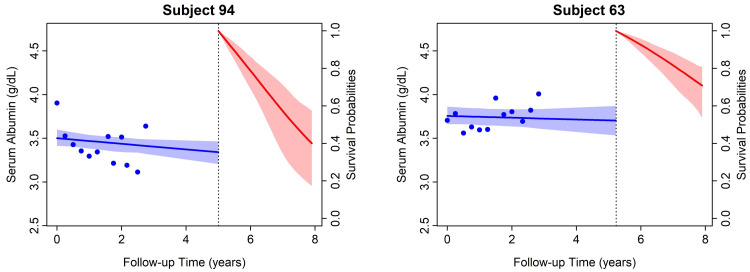
Conditional survival predictions derived from the joint longitudinal and survival modeling from two patients Conditional survival predictions derived from the joint longitudinal and survival modeling from two patients; one without (right panel: subject 63) and one with (left panel: subject 94) significant negative trends of the serum albumin. Higher all-cause mortality probability was observed for the patient with a progressive decline of the albumin levels. The other patient showed no progression throughout the study period (right panel). The solid blue and red lines with %95 confidence intervals represent fitted mean trajectory and survival probability, respectively.

Figure 1 depicts case investigations showing the use of the joint longitudinal-survival model. The model measured the likelihood of all-cause death based on two patients’ longitudinal albumin values. The slope of the albumin in subject 94 was steeper than in subject 63. The subject with a flatter slope (right panel) has better-expected survival chances than the subject with a faster rate of albumin decline (left panel). The crude and multivariate time-dependent Cox results and joint models with a hazard ratio (HR) estimates for the relationship between all-cause mortality and two longitudinal measures of albumin (level and slope in joint models) adjusted for the different baseline variables are shown in Table 2.

**Table 2:** Associations between serum albumin trajectories and the all-cause mortality in hemodialysis patients Hazard ratios (HRs) and 95% confidence intervals (CIs) are presented per one-unit annual increase in albumin estimated by time-dependent Cox model. They are also presented per one-standard deviation annual increase in albumin for level parametrization, and per one-standard deviation annual increase in the rate of decrease (more negative slope) in albumin for slope parametrization, estimated by joint longitudinal-survival model. The model links linear mixed effect (LME) models for the trajectories of the biomarker with Cox proportional hazard models for the time-to-event data. Crude model: Cox model unadjusted, LME model adjusted for sampling time; Model 1: Cox and LME models adjusted for age, sex; Model 2: Cox and LME models adjusted for Model 1 plus body mass index, hemoglobin, ferritin, and dialysis vintage; Model 3: Cox and LME models adjusted for Model 2 plus creatinine, single-pool Kt/V, phosphorus, parathyroid hormone, and C-reactive protein; Model 4: Cox and LME models adjusted for Model 3 plus vascular access type, presence of diabetes mellitus, hypertension, cardiovascular disease, and other comorbidities.

	Time-dependent Cox model		Joint model (level)		Joint model (slope)	
	HR (%95 CI)	p-value	HR (%95 CI)	p-value	HR (%95 CI)	p-value
Crude model	0.32 (0.19 – 0.55)	<0.001	0.71 (0.56 – 0.89)	0.006	2.92 (1.58 – 5.42)	<0.001
Model 1	0.34 (0.20 – 0.59)	<0.001	0.73 (0.64 – 0.85)	<0.001	2.61 (1.24 – 7.29)	0.008
Model 2	0.37 (0.22 – 0.62)	<0.001	0.76 (0.66 – 0.88)	<0.001	1.82 (1.18 – 3.12)	0.001
Model 3	0.39 (0.23 – 0.66)	<0.001	0.76 (0.65 – 0.89)	0.002	1.70 (1.13 – 2.69)	0.010
Model 4	0.41 (0.24 – 0.70)	0.001	0.78 (0.66 – 0.92)	0.008	1.63 (1.08 – 2.84)	0.023

Using the time-dependent Cox model, higher albumin levels were associated with lower mortality risk in the crude model (HR, 0.32; 95% CI, 0.19-0.55; p<0.001). Adjusting for all confounders in the multivariate model did not change the prognostic effect of albumin on survival time (HR, 0.41; 95% CI, 0.24-0.70; p<0.001). Crude joint model for current value (level) parameterization, in which longitudinal submodel was adjusted for sampling time, and survival submodel was adjusted for baseline albumin levels, showed that annual 1-SD increase in albumin level was associated with a decreased hazard of all-cause mortality (HR, 0.71; 95% CI, 0.56-0.89; p<0.006). In a multivariate model, significance was robust to additional adjustments for baseline clinical features and laboratory biomarkers. A yearly 1-SD increase in the rate of albumin decline (faster decliners/more negative slope) was associated with a greater risk of all-cause death (HR, 2.92; 95% CI, 1.58-5.42; p<0.001). The impact of slope on mortality was attenuated but remained significant after all variables were included in the joint model (HR, 1.63; 95% CI, 1.58-5.42; p=0.023) (Table 2).

## Discussion

In this observational cohort study of hemodialysis patients, we identified that a decline in serum albumin over time or a faster drop (higher negative slope) in its levels during the follow-up was related to a greater risk of all-cause mortality applying a time-dependent Cox proportional hazards and joint longitudinal-survival model. The analyses covered all available albumin measurements for hemodialysis patients having routine care in our outpatient dialysis unit, and therefore provide convincing evidence for the convenience of albumin trajectories as potential indicators for patient prognosis. In multivariate models, adjusting for additional potential confounders displayed no substantial influence on this relationship, arguing that the association is independent of these possible confounding variables. The data suggest that temporal alterations in serum albumin play a crucial role in caring for hemodialysis patients at risk. Closer monitoring might give a chance for specialists to appreciate the focus on preventive approaches and reduce the tendency of potential complications.

Albumin levels were a strong predictor of death in several studies in patients with chronic kidney disease. An earlier report with a 1.25-year follow-up found that initial blood albumin levels were a powerful indicator of death in chronic HD patients [11]. A two-year observational study found a significantly increased all-cause and cardiovascular mortality with trimonthly varying serum albumin values [13]. Zitt et al. found an association between time-varying serum albumin and all-cause mortality using the time-dependent cox model in 235 incident HD patients [14]. Another 10-year study found that the low baseline blood albumin level was an independent prognostic factor of death in incident dialysis patients [23]. A recent study investigated the association of two-year serum albumin levels in a longitudinal three-year period by utilizing the serum albumin to reach a rate of 3.5 g/dL and the time-averaged albumin level with mortality. The study showed that HD patients who approached the target serum albumin levels more frequently and had higher time-averaged albumin levels over two years had a survival advantage in the subsequent three-year period [15]. Our findings correspond to previous studies highlighting the importance of time-dependent albumin, but it also provides additional relevant information that its gradient (slope) also is correlated with all-cause mortality risk. We can assume that there is a rapid increase in the intensity of already existing inflammation and oxidative stress in these patients over time.

A time-to-event study with a particular biomarker is needed for seeing individual risk. However, it would give limited information on a disease's clinical history. Heretofore, the predictive value of serum albumin was outlined by changes in a single evaluation, mean levels over a limited period- or time-varying levels handled in the Cox model. Nevertheless, applying a baseline exposure implies that the predictor stays unchanged over the research process, which would be uncommon in long-term studies. The Cox model with time-dependent predictors, on the other hand, takes into account variations in levels of exposure throughout the study. The model assumes that time-dependent covariates are measured without mistake. Moreover, it admits that the value of a covariate at a later time point is unaffected by the event. The second argument does not apply to endogenous variables (clinical biomarkers) since a frequently observed marker such as albumin is directly connected to the mortality pathway [24,25].

As a result, we utilized a combined modeling method to explore the relationship between a longitudinal biomarker trajectory and a time-to-event outcome [26]. We hypothesized that serial serum albumin levels throughout the study period with its rate of change (slopes) would be related to all-cause mortality and useful for patient monitoring and prognostic prediction. Contrasting prior research, ours emphasizes the importance of assessing albumin levels as a function of time using a slope parameterization (decline rate). This issue is addressed by the joint modeling technique, which assumes that the outcomes were computed with an error. Furthermore, adjustment for baseline values has been proposed to minimize the effect of regression to the mean, and the longitudinal submodel with the random intercept term fulfills this function [27]. Last, a joint model is appropriate if repeated data have been collected at various intervals and are unequal in number amongst participants [18].

The advantage of repeated assessments is that a physician can use biomarker levels to assess the probability of adverse outcomes and may change the therapy before an event happens in the future [24]. Our results must be evaluated in light of the study's shortcomings. To begin, residual confounding or other unmeasured inflammation-related variables may have contributed to the emergence of the clinical result. Second, the reasons for hypoalbuminemia in our investigation were unknown. Some factors may have contributed to hypoalbuminemia, including residual urine with proteinuria, hypervolemia, and liver problems. Finally, because the cohort originates from people living in a particular geographical location, the conclusions may not be generalized to different groups.

## Conclusions

Overall, our finding suggests that temporal decreasing of patient-specific albumin levels could predict health outcomes in individuals with incident HD. Patients with a rapidly decreasing rate of albumin over time were more likely to die than those with a steady or slowly declining rate. These patient-specific temporal patterns have the potential benefit, which could be employed for individualized prognosis and therapy plans.
